# Comparative assessment of annotation tools reveals critical antimicrobial resistance knowledge gaps in *Klebsiella pneumoniae*

**DOI:** 10.1038/s41598-025-24333-9

**Published:** 2025-11-18

**Authors:** Kristina Kordova, Caitlin Collins, Julian Parkhill

**Affiliations:** 1 Department of Veterinary Medicine, University of Cambridge, Cambridge, UK; 2 UK Health Security Agency, London, UK; 3 NIHR Health Protection Research Unit in Genomics and Enabling Data, University of Warwick, Warwick, UK

**Keywords:** Computational models, Data processing, Databases, Genome informatics, Machine learning, Sequence annotation, Software, Antimicrobials, Bacteria, Microbial genetics, Pathogens, Microbial genetics

## Abstract

Bacterial antimicrobial resistance (AMR) poses a significant public health threat. The increase of both global awareness and affordable whole genome sequencing has yielded an ever-growing collection of bacterial genome sequence datasets and corresponding antibiotic resistance metadata. This enables the use of computational techniques, including machine learning (ML), to predict phenotypes and discover novel AMR-associated variants. With the great variety of resistance mechanisms to interrogate and the number of datasets that can be mined, there is a need to identify where novel AMR marker discovery is most necessary. Multiple databases and annotation pipelines exist to annotate AMR variants known to be associated with resistance to specific antibiotics or antibiotic classes, however, the completeness of these databases varies, and for some antibiotics, even the most complete databases remain insufficient for accurate classification. Here, we build predictive ML models using only those known markers, which we call “minimal models” of resistance. We predict the binary resistance phenotypes of 20 major antimicrobials in the genomically diverse pathogen Klebsiella pneumoniae, allowing us to identify their shortcomings in phenotype prediction, thereby highlighting opportunities for novel marker discovery. We provide a critical review of the differences in annotation tools and databases commonly used in bacterial AMR studies, and outline guidance for the establishment of a standard dataset for the development and benchmarking of ML models of AMR.

**Supplementary Information:**

The online version contains supplementary material available at 10.1038/s41598-025-24333-9.

## Introduction

As whole-genome sequencing and high-performance computing costs decline, it has become common to utilise increasingly complex in silico approaches to predict the antimicrobial resistance (AMR) profile of bacterial isolates. Studies have sought to incorporate genetic variation across the entire genome through the use of known AMR markers, single-nucleotide polymorphisms (SNPs), k-mers or unitigs, each providing ever more comprehensive coverage. With the increased use of machine learning (ML), the complexity of prediction algorithms has also soared, building on simpler linear models to develop ensemble and deep learning models, able to disentangle nonlinear interactions between variants. This has led to the identification of many potential novel AMR variants^1^. However, alongside essential discoveries, many of the novel hits can be spurious due to the rise of high dimensionality and feature correlation challenges as more comprehensive genomic variation is being analysed. Hence, a question arises as to whether increased computing costs and analysis time will always yield biologically meaningful improvements in classification and variant discovery. This highlights the need for identifying antibiotics and antibiotic resistance mechanisms for which discovering new AMR variants is truly necessary, as well as the establishment of quality benchmarks using only known AMR determinants, to identify whether complex whole genome models lead to significant improvement in phenotype prediction accuracy.

We propose that a “minimal model” of resistance, using only known resistance determinants in the most parsimonious way, can be utilised to demonstrate where discovery of potential novel AMR variants is most necessary, and establish reliable references for whole genome-based ML model performance. We implement this approach using the known repertoire of AMR genes and mutations, drawn from public databases of resistance determinants for a particular antibiotic or class of antibiotics, to build a predictive model using ML. In contrast to comprehensive models, these minimal models are highly computationally efficient, as they utilise the minimum necessary set of features from rapid annotation tools and curated databases^2,3^. The identification of knowledge gaps in known AMR mechanisms is likely to be most relevant in bacteria with open pangenomes, which acquire novel variation more rapidly, such as the genome of *Klebsiella pneumoniae*^4^. This bacterium has been shown to play a pivotal role in amplifying and shuttling resistance genes across Enterobacteriaceae, hence, discoveries in *K. pneumoniae* could have implications beyond this species^5^. By focusing on well-characterised resistance genes in the diverse *K. pneumoniae* population, coupled with predictive machine learning models, we aim to determine where the minimal model approach significantly underperforms, pointing to the need for the discovery of new AMR mechanisms or variants.

The choices of an annotation tool and reference database are a major determinant of the performance of a minimal model. Multiple databases and computational tools have been published, each curated to enable the annotation of bacterial genomes against known AMR markers. Databases include The Comprehensive Antibiotic Resistance Database (CARD)^6^, ResFinder, PointFinder^7^, ResFams^8^, ARDB^9^, UNIPROT^10^, and others. While all databases incorporate a list of genes and gene families, only some extend to species-specific point mutations as well, such as PointFinder and the database associated with the annotation tool AMRFinderPlus^11^. Each database has also been curated with different rules, resulting in differences in antimicrobial resistance gene (ARG) content^12^. Some have focused on stringent validation, such as CARD^6^, while others have included an array of variants predicted to have an impact on phenotype with high confidence, such as DeepARG^13^.

Currently, there are more than 18 commonly used species-agnostic open-source command line tools designed to rapidly identify the presence of genes and/or mutations^14^. Annotation tools such as AMRFinder^2^ and its newer version AMRFinderPlus^11^, Resistance Gene Identifier^6^, and Abricate^15^ rely on the previously listed databases to match genomic sequences against the reference and annotate the presence of AMR variants in each sample. Furthermore, species-specific annotation tools have also been developed, which match sequences against the variation reported in the bacteria of interest. These tools have the potential to yield less spurious and more concise gene matching. For example, the tool Kleborate is designed specifically to catalogue the variation in the bacterium *K. pneumoniae*^16^. Other pipelines include TBprofiler^17^, specialising in annotations of *Mycobacterium tuberculosis* and Mykrobe^18^, focusing on multiple selected species. These pipelines differ in supported inputs, search algorithms, parameterisation and output formats. The complexity of underlying methodologies also varies significantly, which results in different granularity and quality of annotation. For example, the commonly used Abricate uses the NCBI database by default but only covers a subset of what AMRFinderPlus encompasses, resulting in the inability to detect point mutations, as well as some genes. Hence, an informed choice of annotation tool and database can be difficult and potentially case-specific, inevitably leading to variation in the performance of minimal models^19^.

Here, we use minimal models to compare the completeness of gene annotations produced by eight commonly used annotation tools, applied to assembled genomes of *K. pneumoniae*. By doing so, we aim to highlight antibiotics where known mechanisms do not fully account for the observed variation in resistance, and therefore where ML-enabled marker discovery will be most valuable. To generate phenotype predictions from these collections of annotated genes, we used ML models of varying complexity based on the presence/absence of the annotated markers. The performance of each tool is then explained in the context of genes that receive high importance scores by models during prediction, showing which antibiotics emerge as avenues of interest, either as well-studied or underperforming in classification based on known mechanisms.

## Methods

### Data collection and pre-processing

For this analysis, we sought to explore a dataset which is becoming more commonly used as a standard source of bacterial samples - the Bacterial and Viral Bioinformatics Resource Centre (BV-BRC) public database^20^. The whole genome sequences of 18,645 *K. pneumoniae* samples filtered for good-quality assemblies were obtained. The length of genomes averaged 5.6Mbp, and assemblies consisted of up to 1000 contigs. The majority of deposited samples were collected from clinical studies, from both patients and hospital environments, spanning 66 countries. Outlier genomes with more than 250 contigs and lengths of more than 6.4 Mbp and less than 4.9 Mbp base pairs were excluded from downstream analysis due to low quality and possible contamination. To identify genomes from other species, all sequences were species- and MLST-typed using Kleborate v2.2.0^16^. 125 samples were removed, matching to species *K. quasipneumoniae subspecies quasipneumoniae*,* K. quasipneumoniae subspecies similipneumoniae*,* K. quasivariicola* and *K. variicola subspecies variicola*.

**Fig. 1:**
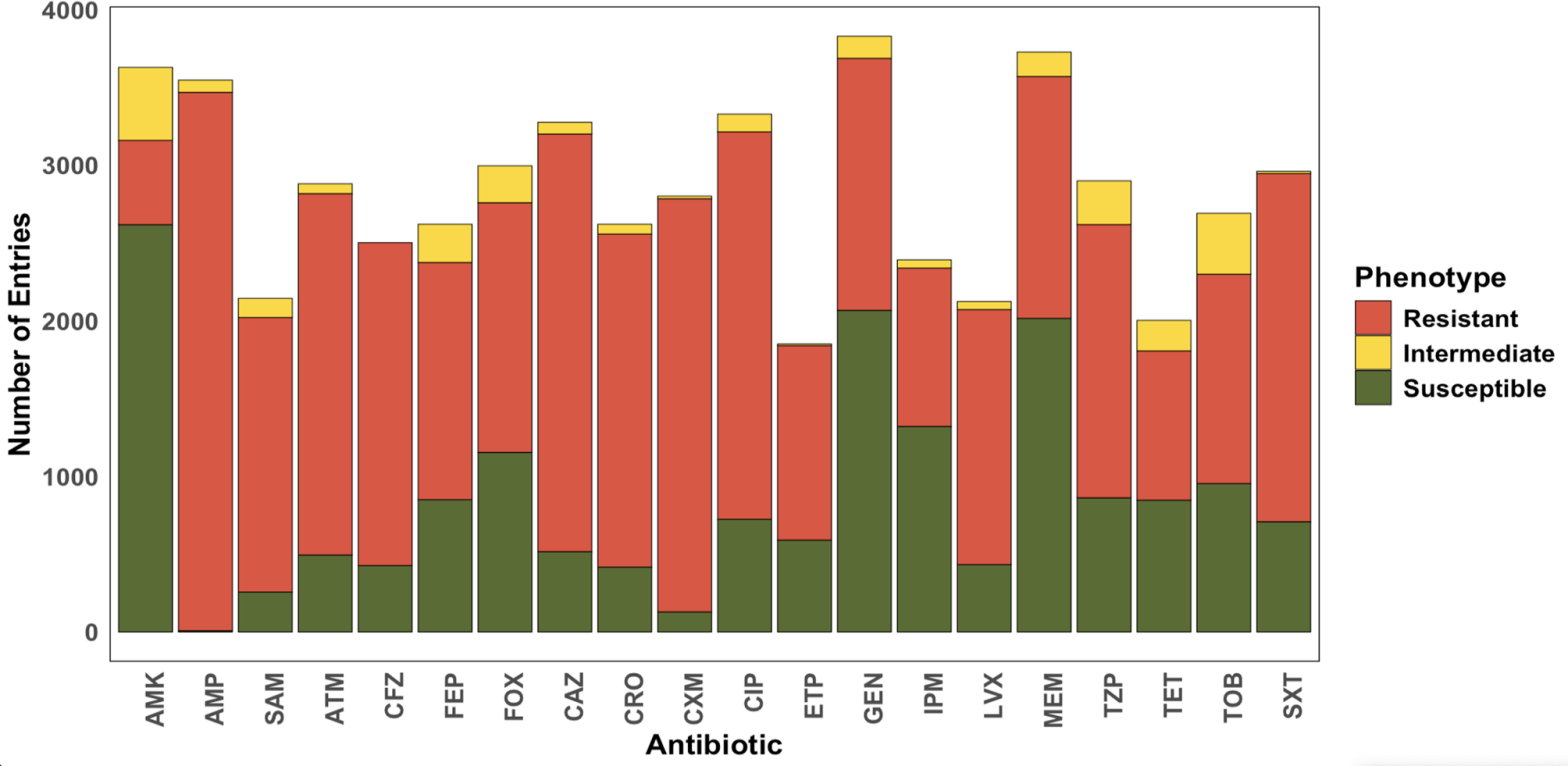
Distribution of phenotypes across antibiotics.

Antimicrobial resistance data for 76 antibiotics tested by clinical phenotyping were available for 4,976 of the BV-BRC-associated genomes, grouping into 15 antibiotic classes. Antibiotics for which data was available for less than 1800 samples were excluded from analysis, as the low sample size is expected to yield spurious results. This led to a further reduction of the number of genomes to 3,751. Antibiotic abbreviations are defined in Supplementary Table 1. Although MIC prediction has higher clinical relevance, binary resistance profiles tend to yield higher prediction performance and be more suitable for basic ML architectures^21^. We acknowledge that the resistance breakpoint, as reported by the European Committee on Antimicrobial Susceptibility Testing (EUCAST) and Clinical and Laboratory Standards Institute (CLSI) could have changed through the years and re-conversion of the MIC might be beneficial. However, not all studies reported MICs, so we utilised the resistance labels as provided by the BV-BRC database to allow for consistency with the standard database. Phenotypes across antibiotics are shown in Fig. 1.

**Table 1:** Eight AMR gene annotation tools which were compared in this analysis, describing their target species, input, supported databases and output format of the relationship between the gene and the associated AMR by either antibiotic or antibiotic class.

Tool	Organism	Input	Database	Output
ABRicate	Agnostic	Contigs	NCBI, CARD, ARG-ANNOT, Resfinder, MEGARES, EcOH, PlasmidFinder, Ecoli_VF and VFDB	Gene-to-class
AMRFinderPlus	Agnostic	Protein and/or assembled nucleotide sequences	AMRFinderPlus	Gene-to-class
DeepARG	Agnostic	Metagenomic data, Short reads, Long sequences	DeepARG-DB	Gene-to-class
ResFinder	Agnostic	Contigs	ResFinder, PointFinder, DesintFinder	Gene-to-antibiotic
RGI	Agnostic	Contigs	CARD	Gene-to-antibiotic
SraX	Agnostic	Contigs	AMR DB	Gene-to-class
StarAMR	Agnostic	Contigs	ResFinder, PointFinder, and PlasmidFinder	Gene-to-antibiotic
Kleborate	*Klebsiella pneumoniae*	Contigs	Kleborate	Gene-to-class

### Sample annotation and minimal gene subsets

We reviewed 19 popular annotation tools, discussed by previous studies (Supplementary Table 2), of which eight were applicable to the analysis of assembled *K. pneumoniae* genomes (Table 1)^14^. After review, the available samples were annotated using Kleborate, ResFinder, AMRFinderPlus and DeepARG against their default database settings, RGI, SraX and Abricate against the CARD database, and StarAMR against ResFinder. Virulence gene hits were excluded from these annotations. Different tools annotated samples in either gene-to-antibiotic or gene-to-class relationships. To compare the performance of minimal models predicting resistance to antibiotic classes with resistance to individual antibiotics, we further acquired a list of genes corresponding to each of the 19 antibiotics of interest from the stringent CARD ontology database. The database encompasses gene-to-antibiotic and mutation-to-antibiotic relationships, where these have shown an experimental increase in MIC. Subsets of genes-to-class and genes-to-antibiotic were used for downstream ML model fitting. In cases where susceptibility testing was carried out for combination therapies, the genes for both classes were included in the minimal subset (e.g. the gene set for amoxicillin-tetracycline was a combination of the sets for amoxicillin and tetracycline). Where genes were annotated as multi-drug, they were included in all subsets, as they often marked efflux pumps and porins relevant across antibiotics. These were not subset further as their specific function in relation to each class or antibiotic is difficult to disentangle or is not fully known. To further format annotations as features for the prediction models, positive identifications of resistance genes or gene variants were formatted as a feature presence/absence matrix **X**_p×n_∈ {0,1}, where *n* denotes the number of unique AMR features and *p* represents the number of samples. **X**_ij_ = 1 if the AMR feature is present in this sample and **X**_ij_ = 0, otherwise.

### Machine learning models

The performance of two types of predictive models was compared when using these generated marker subsets as features, namely logistic regression with L1 and L2 regularisation (Elastic Net) and the Extreme Gradient Boosted ensemble model (XGBoost). These were chosen due to their interpretability and scalability, as well as their usually high accuracy. Their specific characteristics and parameters are described below. The dataset was split into 70% training and 30% testing, using a stratified fivefold cross-validation without hyperparameter tuning. The performance of the models was scored by observing the sensitivity, specificity and area under the receiver operating characteristic curve (AUC). Due to imbalances in the relative number of resistant and sensitive isolates across antibiotics, we find that AUC appeared as a more informative reporter of the model fit than the more commonly used balanced accuracy. The target labels were binarised so ‘resistant’ also included ‘intermediate’ samples, as reported by the BV-BRC database. Model code is available at: https://github.com/kristinakordova/minimal_models.

To explain differences in model performance, we scored the names and number of genes used for prediction by each annotation tool. Where the ARG determinants of classification performance were not immediately obvious, we used Shapley values to explain the XGBoost model and logistic regression coefficients to identify features which received high-importance scores in classifications. Shapley values were extracted using the SHAP python package and TreeExplainer with default parameters^22^. They represent the contribution of each feature to the classification of each sample in the XGBoost models. Features were ranked in accordance with a single Shapley value, derived for each feature in the corresponding model using the mean of the absolute values for the Shapley values across samples, as per the default use of the package. Important feature sets were then compared between tools that showed high and low performance for the antibiotic of interest.

### Elastic net

Elastic Net is a penalised linear regression model that includes both the L1 (LASSO) and L2 (Ridge) penalties in the loss function during training. The L1 penalty shrinks the coefficients towards zero, which removes many predictors from the model. The L2 penalty instead minimises the Euclidean norm of the coefficient vector but typically produces models that use all the predictors. The model assumes a linear relationship between input variables and outputs, and is therefore not capable of identifying potential inter-dependencies between multiple variants and the phenotype. Elastic Net is believed to manage dependent variables, expected due to the strong linkage disequilibrium in bacterial populations^23^. The model was tested using α = 0.01 and a switching parameter of 0.5, indicating the model performs as a Ridge selection and as LASSO. Weight balancing between resistant and susceptible classes was not included in the final report. The model was run using the python package sklearn v4.1.3.

### XGBoost

XGBoost is a tree ensemble method that provides the advantage of decorrelating sets of trees through feature and sample subsampling. Studies have reported that XGBoost is able to manage the multicollinearity of small feature sets^24^. It allows for feature interaction, as features which form the follow-on nodes on each decision tree depend on the features in the previous nodes. Hence, this approach advances the Elastic Net by allowing for an extra level of statistical and potentially biological complexity. The XGBoost model was trained using 30 trees with a depth of 3 to reduce the possible overfitting of the small minimal models. Weight balancing between target classes was not included to yield comparable results to the Elastic Net model. The Random Forest model was run using the python xgboost package v2.1.1.

### RGI

The RGI pipeline is versatile in its supported input formats, covering genomes, genome assemblies, metagenomic contigs, k-mers, reads or proteomes^6^. It carries out gene prediction using Prodigal, homolog detection using DIAMOND, and Strict significance based on CARD curated bit score cut-offs^25,26^. The tool outputs annotations in a gene-to-antibiotic relationship. We used RGI v.6.0.3.

### ResFinder

The ResFinder pipeline utilises BLAST and offers a choice of annotation databases including ResFinder and PointFinder^7^. ResFinder curates horizontally acquired ARGs and outputs annotations in a gene-to-antibiotic relationship. PointFinder annotates point mutations in ARGs in a gene-to-class manner. ResFinder is one of the few tools that also predicts the phenotype of *K. pneumoniae* samples for specific antibiotics. We therefore performed a comparison between our ML phenotype prediction and the rules-based prediction of ResFinder to compare their results. We utilised ResFinder v.4.1.5, which was last updated in 2021^7^.

### AMRFinderPlus

AMRFinderPlus is the updated version of AMRFinder, released by NCBI^11^. The pipeline has the advantage of being able to detect the presence of both genes and point mutations against its own curated database. Sequences are matched using BLAST and Hidden Markov Models. AMRFinderPlus v.3.12.8 was used.

### DeepARG

Based on a pre-trained multiclass deep learning model, DeepARG is able to detect AMR genes from both short and long sequences^13^. The tool collates and curates the databases ARDB, CARD and UNIPROT, significantly increasing the diversity of available known AMR sequences. The prediction is carried out by generating a dissimilarity matrix between sequences from predicted genes and known ARGs using DIAMOND, instead of BLAST, which reports higher speed. The resulting output is genes associated with antibiotic classes. The classification is reported to differ in fidelity between classes, with major antibiotic classes being well classified and classes with fewer known genes and multi-drug resistance gene classes performing poorly. Here, genomes were annotated using DeepARG v.1.0.2. against the corresponding database for this software version. Annotations with a probability greater than 80% were included in the analysis. Genes annotated as ‘multi-drug’ were included in all subsets.

### Abricate

The Abricate tool is designed to detect ARGs from assembled contigs by aligning genomes to reference databases using BLAST^15^. The user can select the reference database from an extensive list, including NCBI, CARD, ARG-ANNOT, and others. Hence, an informed user choice could be necessary to select the relevant database. Furthermore, the tool encompasses only gene presence but not point mutations. Abricate v1.0.0. was used to annotate all available genomes against the CARD database.

### Kleborate

Kleborate uses assemblies and outputs annotations of resistance genes by drug class, alongside other quality control metrics^16^. Nucleotide BLAST is used against the CARD database, as well as other genes and alleles which have been clinically demonstrated to impact MIC profiles in the *K. pneumoniae* species complex. As well as genes with high identity match, the tool also annotates truncated and spurious hits, which were included as features if associated with the antibiotic class of interest. This yields a high number of annotations, as we conserve genes with lower identity matches as separate features from genes with high identity matches and rely on the downstream models to assign the appropriate informativeness. We used Kleborate v.2.2.0 rather than the newer v.3.0.0 due to software compatibility.

### SraX

The sraX tool utilises a locally compiled AMRDB connecting to CARD as the default database^26^. A homolog search is performed by alignments to the database, facilitated using DIAMOND. The tool annotates genome assemblies and provides a gene-to-class level annotation. The sraX v.1.5 was used, and annotation identities and sequence matches were set to 95% and 90%, respectively. Genes annotated as ‘multi-drug’ were included in all subsets.

### StarAMR

StarAMR queries the ResFinder, PointFinder and PlasmidFinder databases using a BLAST-based approach to scan bacterial genome contigs for ARGs and mutations^27^. It compiles a summary report of genetic mechanisms and a predicted antibiogram based on a gene-to-antibiotic key. We used the only release of StarAMR – v.0.10.0, and the corresponding database updates as of January 2023.

### Integrated dataset

An integrated annotation dataset was created by merging annotations from different tools. We first merged all datasets for each antibiotic and removed features with identical presence/absence patterns across tools. Gene names were then indexed with the name of the annotation tool they originated from, to distinguish between genes with identical names but different presence/absence patterns between annotation tools. These differences likely arise from differences in sequence databases or alignment tools used by each tool and could be of varying quality. Hence, the model was allowed to select the most informative annotation of these genes. The integrated dataset was then used in downstream analysis analogously to minimal gene and variant sets from each tool.

### Other annotation tools

Reviews and consortia, such as the Public Health Alliance for Genomic Epidemiology (PHA4GE), have additionally noted a few more annotation tools which were not used for the construction of our minimal models. The popular ARIBA^28^, AMR++^29^ and SRST2^30^ pipelines were not used as they use raw reads, not genome assemblies. GROOT^31^ and KmerResistance^32^ were excluded as they use metagenomic data or poorly sequenced genomes, respectively. Similarly, fARGene^33^ utilises fragmented sequences and pre-trained AMR class predictive models to predict full-length sequences, however, models need to be run individually, which was not feasible for our analysis. Mykrobe^18^ was not included as it does not support models of resistance for *K. pneumoniae*. c-SSTAR^34^ and ResFams^8^ were not included due to installation challenges.

## Results

**Fig. 2:**
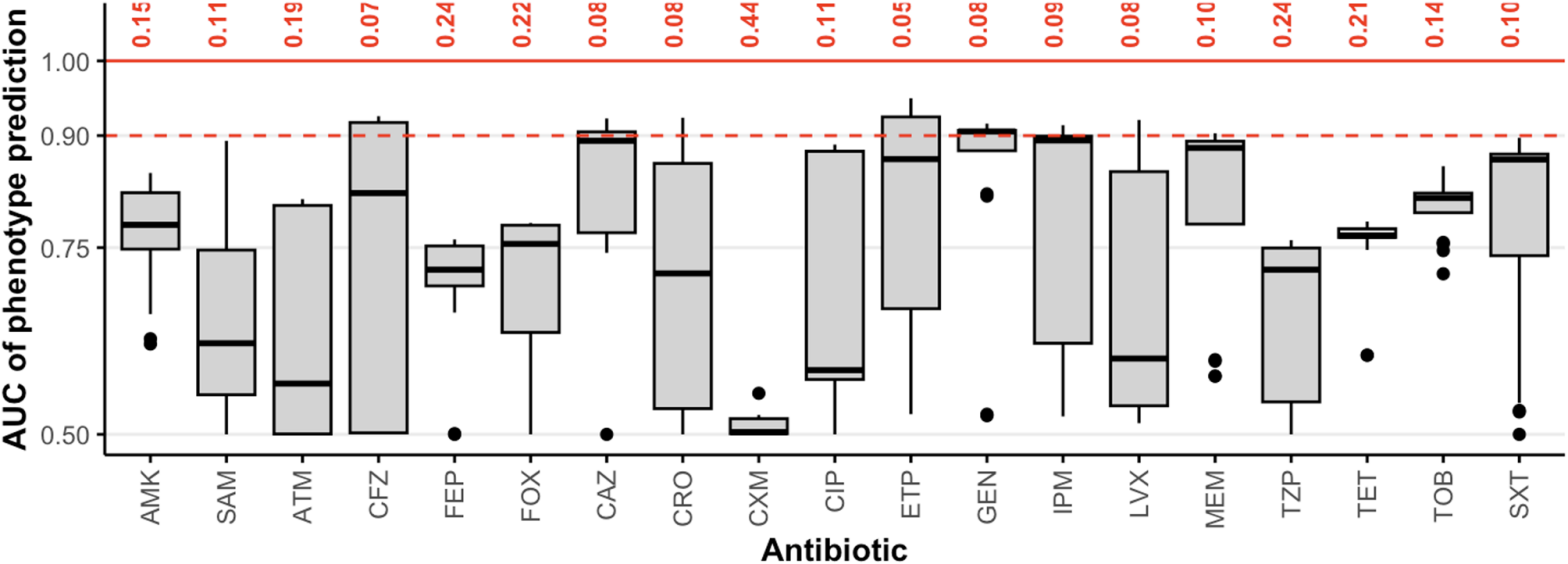
Summary of the minimal model performance across antibiotics, showing average performance across annotation databases. The difference between the highest AUC value achieved and one (complete predictive success) is indicated in red for each antibiotic. The red dashed line highlights the threshold of 0.9, reached by some models using features from certain annotation tools .Outlier predictions AUC value of 0 are excluded as they result from having no annotated features for the respective antibiotic.

The AMR-associated genes and variants identified by the eight commonly used annotation pipelines listed in Table 1a were used as features in ML models to predict the drug resistance phenotype of each sample. The aim of this analysis was to determine where these minimal models of drug resistance, containing antibiotic- or antibiotic class-specific AMR markers identified by each tool, fail to accurately predict drug resistance. This comparison serves two purposes. First, in Fig. 2, we show which antibiotics remain poorly predicted across all AMR databases and annotation approaches, indicating the need for further study. Second, in Table 2, we reveal that substantial variations in predictive performance exist within as well as across antibiotics, depending on which AMR database and annotation tool is used to define the set of known AMR markers in the minimal model.

The ability to predict AMR status from known AMR markers varied considerably across the 19 antibiotics examined. Figure 2 displays the predictive performance of the minimal model for each antibiotic across all eight annotation tools. Low AUC values indicate where predictions are most discordant from the observed binary resistance phenotype for a particular antibiotic. While AUC values over 0.9 are attained for some approaches for seven of the 19 antibiotics using specific annotations, such as cefazolin (CFZ), ceftazidime (CAZ), ceftriaxone (CRO), ertapenem (ETP), gentamicin (GEN), imipenem (IPM), levofloxacin (LVX), none of the 19 antibiotics achieves an average AUC across annotations above this level. Resistance to gentamicin (GEN), trimethoprim/sulfamethoxazole (SXT), tetracycline (TET) and tobramycin (TOB) was on average well predicted by most tools, while resistance to cefuroxime (CXM), ampicillin/sulbactam (SAM) and piperacillin/tazobactam (TZP) was most often predicted poorly. While performance appears to vary greatly, AUC values for gentamicin (GEN), tetracycline (TET) and tobramycin (TOB) appeared most consistent across tools.

**Table 2:**
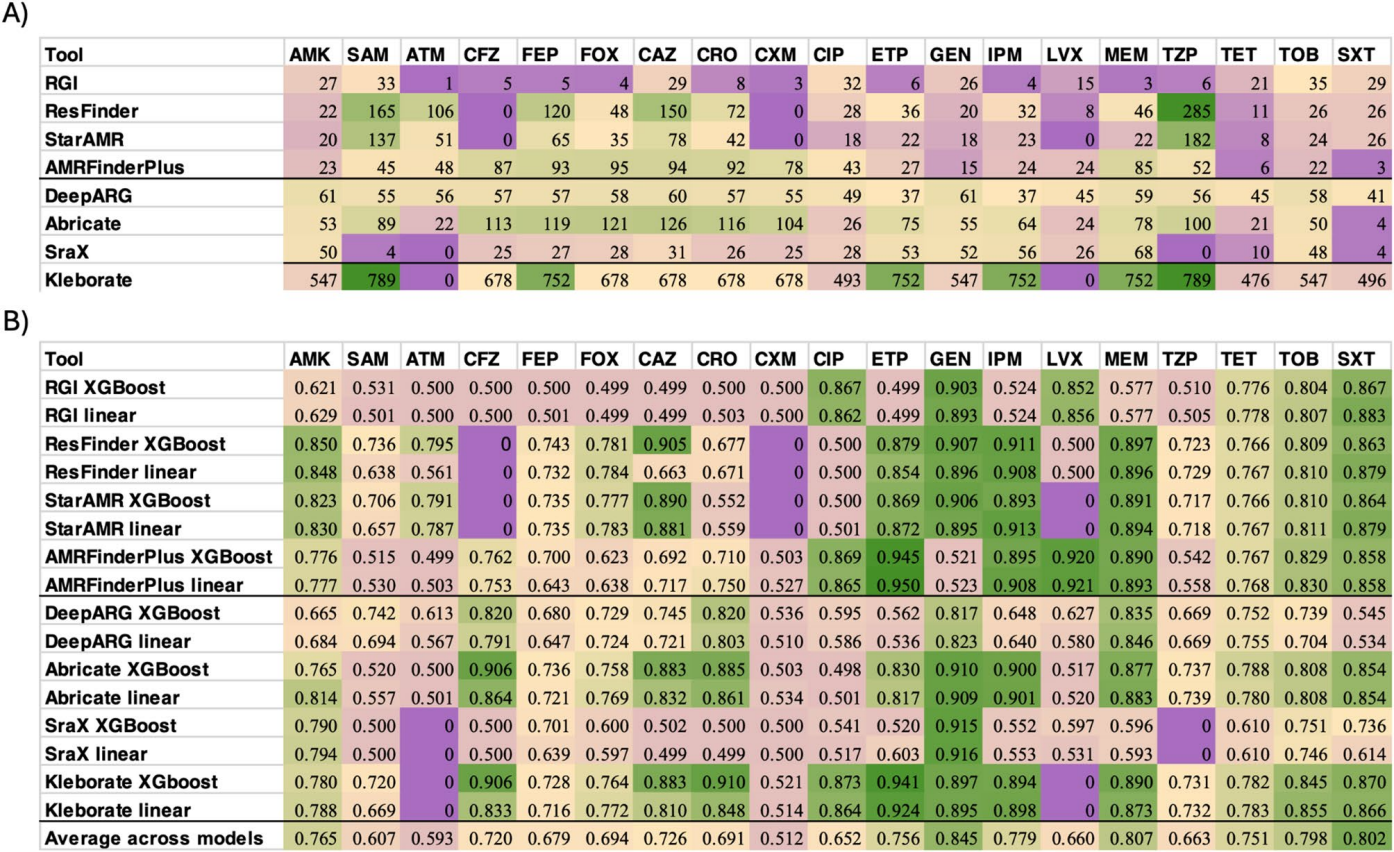
Summary of the annotation and prediction results across antibiotics and annotation tools. (A) number of AMR-associated genes and variants identified by each tool for each antibiotic or antibiotic class, and (B) individual AUC values across all annotation tools. Results are coloured from dark purple (low values) to green (highest values). Bold lines divide tools carrying out gene-to-antibiotic prediction from gene-to-class prediction. Gene number for kleborate is lined out and coloured separately due to the Stark difference in gene number. The row average across models indicates the average prediction performance across models and annotation tools for each antibiotic. The calculation excludes values of zero, as they are a result of missing annotation and not poor performance.

The number of annotated AMR genes and variants identified differed significantly between annotation tools and across antibiotics (Table 2a). RGI was shown to be the most conservative approach across all antimicrobials, identifying the fewest AMR markers across most antibiotics, likely due to the underlying annotation methodology and the gene-to-antibiotic relationship of variants. On the other hand, ResFinder annotated more hits across antibiotics, but failed to identify ARGs associated with resistance to cefazolin (CFZ), levofloxacin (LVX) and cefuroxime (CXM). AMRFinderPlus, DeepARG and Abricate found a higher number of annotated genes across most antibiotics. The number of genes identified by DeepARG appear mostly similar across antibiotics, as genes annotated as multi-class were included in all antibiotic-specific subsets. Kleborate typically discovered the greatest number of ARGs, as the tool annotates truncated proteins and genes with different percentage identities, which we kept as distinct features. No associated genes were identified by this tool for aztreonam (ATM) and levofloxacin (LVX). Major differences were observed in the phenotype prediction performances of the eight tools by the two ML models (Table 2b, Supplementary Table 3). While in most cases the higher number of predictive AMR markers resulted in higher performance, exemplified by lower performances in RGI annotations utilising fewer genes, this was not always the case. For example, amikacin (AMK) was best predicted by ResFinder utilising fewer genes and variants than DeepARG, Abricate, Kleborate and SraX. The gene-to-antibiotic tools showed distinct performances, with the RGI showing lower AUC than ResFinder for most antibiotics. This is in line with the fewer ARGs annotated by RGI, which limits the prediction potential, while ResFinder relies on a much wider feature space. All annotated variants per tool and antibiotic are listed in Supplementary Table 4, as well as the Shapley values describing the variants with higher weight in each prediction task.

Significant prediction performance differences were observed for the antibiotics ciprofloxacin (CIP) and levofloxacin (LEV). AMRFinderPlus and Kleborate results contained more granular characterisation of the gyrase genes *gyrA* and *gyrB*, including putative mutations, which likely led to good classification performance. As they are core genes, gyrases were absent in the ResFinder, DeepARG and Abricate annotations, which likely led to the inability to predict the fluoroquinolone resistance profile using these tools. Instead, these tools annotated multiple *qnrB and qnrA* genes.

Resistance to sulfamethoxazole/trimethoprim **(**SXT) was well predicted by AMRFinderPlus by annotating only three genes, namely *sul1*, *sul2* and *sul3*. Although this does not account for trimethoprim resistance, this is likely due to the complete correlation of resistance to trimethoprim and sulfamethoxazole in this dataset, probably due to the historic use of trimethoprim solely in combination with sulfamethoxazole^35^. These genes were also annotated by all other tools as well. Interestingly, while these genes were annotated by DeepARG and Abticate, they were often identified in different samples, compared to AMRFinderPlus, which likely led to the underperformance of these tools. *dfrA* gene variants were also given high importance whenever detected by annotation tools, in line with the nature of resistance to combination therapy. Gentamicin (GEN) resistance was poorly predicted by AMRFinderPlus, which was missing annotations for *aac(3)* gene variants, annotated by all other tools, as well as *armA*. These genes are major determinants of resistance to aminoglycosides and have a high Shapley value for the classification of gentamicin by most tools. The genes were also missing in the amikacin AMRFinderPlus annotation, however, they did not receive a high Shapley value in predictions by other tools. Instead, variants of the genes *aac(6)* and *aph(3’) -VI* were shown to have high importance, which were annotated by all tools.

ML models for aztreonam (ATM), cefepime (FEP), cefoxitin (FOX), piperacillin/tazobactam (TZP), tetracycline (TET) and tobramycin (TOB) performed mostly universally well across tools, despite the large differences in gene numbers. In some cases, such as ampicillin/sulbactam (SAM) and cefuroxime (CRO), the XGBoost model overperformed the Elastic Net predictions. This could be interpreted as arising from the higher model complexity or due to the need to incorporate variant interaction as a strong determinant of resistance. However, as the annotations do not provide a granular and comprehensive genome coverage, the role of interaction effects in these antibiotics needs to be studied further.

Resistance to meropenem (MEM) was poorly classified based on the annotation from RGI, likely due to many missing genes, as this tool detected only three AMR genes for this antibiotic. The underperformance in imipenem (IMP) classification by RGI and SraX was likely due to the missing annotation of *blaKPC* and *ompK*, which were present in all other annotations and marked as important by high Shapley values.

We next sought to identify whether combining features differing between annotation tools might decrease the knowledge gap. We fitted the ML models using an integrated dataset of features from all tools (Supplementary Fig. 1). We observed an improved performance for the antibiotic cefuroxime (CXM), when compared to the best performing minimal model using features from DeepARG. The top Shapley values for the integrated dataset demonstrated that the XGBoost model mostly utilised features from DeepARG, as well as Abricate (*CTX-M-15* and *KPC-1*) and AMRFinderPlus (*blaCTX-M-15*). However, for most other antibiotics the integrated dataset showed comparable or lower AUC than the top performing minimal model. Notably, predictions for piperacillin/tazobactam (TZP) demonstrated significantly lower AUC. The top features in the integrated dataset originated from DeepARG, AMRFinderPlus, Abricate, ResFinder and Kleborate. Features which were missing in the Abricate list were *OmpK45* (Kleborate), *OmpK36GD* (Kleborate), *CTX-M-15* (ResFinder). However, due to the increased number of features and their differences in presence/absence patterns between tools, pinpointing the precise cause of the underperformance is challenging. Furthermore, predictions for ampicillin/sulbactam (SAM), cefepime (FEP), ceftriaxone (CRO), cefazolin (CFZ), ceftazidime (CAZ) and aztreonam (ATM) showed increased variance in prediction quality in the 5-fold-cross validation. This demonstrates that fewer, well-curated features can lead to better ML model performance, as well as validating the utility of using a single annotation tool for minimal model predictions in this dataset.

We also investigated whether curated rule-based prediction might perform better than our minimal models, and explain the apparent knowledge gap. To do this, we compared ML-based prediction using the ResFinder annotations to the rule-based phenotype calls from ResFinder itself. We found that ML models have higher or comparable AUC for most antibiotics, except ciprofloxacin (CIP) and ceftriaxone (CRO), where the rule-based ResFinder phenotype prediction showed higher performance (Supplementary Fig. 2). The lower performance of the minimal model for these antibiotics was predominantly driven by the lower specificity, indicating some model overfitting. The higher performance for other antibiotics was mainly attributed to an improved specificity of the ML-based predictions, as they are generally expected to better call susceptible samples, even in the presence of the resistance markers.

## Discussion

In this analysis, we demonstrate that annotation tools show differences in their annotation patterns and breadth, which further translates to a varying and often low utility for ML-enabled phenotype prediction. This is in line with expectations and confirms previous findings of multiple studies, which demonstrate that the use of known ARGs is insufficient to provide clinical-grade testing, where a maximum error rate of 3% is permitted^12,18,36^. We further demonstrate that no annotation tool is consistently better than the others across all antibiotics, with pipelines such as AMRFinderPlus overperforming in predictions of some antibiotics while underperforming in others. However, annotations with Kleborate showed the most consistent usefulness in ML classifications. This finding is also in line with expectations, as Kleborate is specifically curated to include AMR mechanisms in the bacterium of interest *K. pneumoniae*.

The potential to compare the selected pipelines and their ability to carry out AMR gene detection and prediction was greatly hindered by the lack of standardisation in the output format. This necessitated manual curation of the genes and mutations corresponding to resistance to individual antibiotics, creating an avenue for error. As demonstrated, the AMR genes listed by CARD ontology terms appear difficult to match against annotation pipelines and lead to underperformance in phenotype prediction. The recently developed pipeline hARMonisation has aimed to address this challenge by harmonising the gene names and formats across annotation tools^14^. Its application spans widely across 17 bacteria-agnostic annotation tools but is not adapted to report the association of each gene with resistance phenotype beyond what is reported by individual tools. Hence, a gap remains for the creation of a community standardisation tool for gene annotations and AMR association with individual antibiotics. This will be necessary to enable the true application of minimal models.

Importantly, use of minimal models to collate resistance markers from across databases and pipelines points to clear prediction shortfalls, where known mechanisms do not account for all phenotypic resistance. These shortfalls present opportunities for the discovery of new mechanisms driving resistance in *K. pneumoniae*. In this dataset, we demonstrate consistent underperformance in predictions of resistance to ampicillin/sulbactam, cefepime, cefoxitin, cefuroxime, piperacillin/tazobactam and tetracycline. Interestingly, these findings were not concentrated in a single antibiotic class, which can be attributed to several factors. Firstly, although antibiotics belonging to the same class are similar in function, they nevertheless exhibit differences in chemical structures and belong to different development generations. Hence, an antibiotic class-based approach to prediction (as used in many current tools) is not sufficient to account for the subtleties of differential resistance to antibiotics in the same class. This has also been noted by previous studies which attempted to carry out phenotype prediction^36^. Hence, the use of gene-to-antibiotic annotations might be more appropriate for the use of minimal models for phenotype prediction. Nevertheless, the same gaps were also identified for tools which carry out gene-to-antibiotic predictions, which supports the finding of true knowledge gaps on resistance mechnisms.

In addition, this analysis is challenged by some clear limitations of the annotation tools and the use of genome assemblies, which resulted in the inability to incorporate the effect of ARG copy number into the analysis. Samples in the BV-BRC database were sequenced predominantly using short-read sequencing techniques, resulting in fragmented genome assemblies of many contigs with only a few fully resolved genomes from long-read or hybrid sequencing. As a result, tandem repeats, which may result in gene amplifications, can be collapsed in the process of genome assembly. Tandem gene amplifications are a known mechanism of modulating resistance in gram-negative bacteria, which was impossible to account for in this analysis^37^. ARG copy number is also affected by their carriage on plasmids, which are often impossible to resolve in short-read datasets. Dosage effects have been hypothesised to have a major role in resistance to carbapenems and other antibiotics, hence, incorporation of copy number could lead to an improvement in phenotype prediction^38^.

This analysis is also inevitably biased by the characteristics of the available dataset. We opted to use the *K. pneumoniae* samples available through the BV-BRC database as it has been utilised by multiple previous studies and is emerging as a somewhat standardised dataset of bacterial samples, suitable for model benchmarking^23,39^. The database provides a large and diverse collection of *K. pneumoniae* samples spanning geographies and sampling niches. However, the collection is often biased in favour of one of the resistance phenotypes, as demonstrated in Fig. 1. This poses a challenge for the training of ML models and could render the ARG features irrelevant. Of the antibiotics we studied, cefuroxime appeared most imbalanced, followed by amikacin; however, cefepime, cefoxitin, tetracycline and piperacillin/tazobactam appear more balanced when samples labelled intermediate are converted to resistant. This binarisation of the target label could be a further confounding factor due to changes in MIC breakpoints through time, also noted by Mahfouz et al.^36^. For example, ciprofloxacin CLSI breakpoints for Enterobacteriaceae have increased from the initial S ≤ 0.25, I = 0.5, *R* ≥ 1 to S ≤ 1, I = 2, *R* ≥ 4. Hence, reference to the source studies and manual curation could be beneficial to ensure consistency in MIC values through the re-assignment of binary labels. This was out of the scope of this study as we aimed to utilise the sample cohort as a standardised dataset. Furthermore, the BV-BRC dataset is also prone to label errors related to testing and reporting practices in different geographies. Hence, further analysis is necessary to estimate the signal-to-noise ratio of reported resistance for samples in these subsets, in order to truly pinpoint whether the source of underperformance is insufficient knowledge of the biological mechanisms underlying the observed phenotypes or inaccuracies in the training set.

Once validated as true discovery opportunities, the search for new antimicrobial mechanisms for these antibiotics should include broad variation across the genome, spanning variants in genes as well as variation outside coding regions. In this pursuit, we believe the use of the appropriate ‘minimal model’ will provide an invaluable benchmark, demonstrating whether advancements in complexity lead to significant improvements in prediction performance. Furthermore, their use will enable researchers to pinpoint truly novel variation, versus variants associated with resistance due simply to high correlation. Analysis by Mahfouz et al.^36^ discussed the role of regulatory regions in modifying the roles of multi-subunit efflux pumps and porins in determining phenotype, showing that the removal of multi-drug-associated genes can increase prediction accuracy. As we did not aim to maximise performance but instead point to where knowledge is missing, we did not carry out any further feature engineering or selection beyond subsetting minimal ARG sets. Therefore, we argue that regulatory sites should be characterised as a class of contributors, rather than excluding the mechanisms they influence. Furthermore, as false positive results are also of major clinical concern, including markers of susceptibility in ARG databases might also be beneficial. In this analysis, we relied on the penalties of the Elastic Net and XGBoost models to carry out appropriate feature selection in the context of the prediction task.

## Conclusions

In this analysis, we utilised minimal models of antibiotic resistance in *K. pneumoniae* to identify and quantify a knowledge gap in the known mechanisms of resistance to the antibiotics ampicillin/sulbactam, cefepime, cefoxitin, cefuroxime, piperacillin/tazobactam and tetracycline. The poor predictive performance for these antibiotics demonstrates an opportunity and a pressing need for the discovery of new AMR variants and mechanisms. We also show that resistance to gentamicin, imipenem and ertapenem is well-classified based on the reviewed annotations, hence, new ML models can be benchmarked against this minimal model and are expected to find well-known AMR markers. We utilised eight commonly used AMR annotation tools to identify known mechanisms of resistance in the sample cohort and demonstrated stark differences in the breadth of annotations and prediction potential. Hence, we caution informed use of benchmarks for ML models. We note that the use of minimal models would ultimately be unlocked when resistance gene sets are annotated in a gene-to-antibiotic manner, as opposed to the present gene-to-class annotation provided by most tools, to capture subtleties of differential resistance to antibiotics in the same class. Further work will seek to analyse the noise-to-signal ratio in this dataset and validate the discovery opportunities for further whole-genome ML-enabled mining. Ultimately, the analysis presents a valuable exploration of the BV-BRC dataset as a standardised dataset for benchmarking and developing novel ML models, and specific antibiotics, where they can be validated or used to discover unknown markers.

## Supplementary Information

Below is the link to the electronic supplementary material.

Supplementary Material 1

Supplementary Material 2

## Data Availability

The datasets generated and/or analysed during the current study are available in the ‘BV-BRC Klebsiella pneumoniae AMR Annotations’ repository, https://zenodo.org/records/17313310?token=eyJhbGciOiJIUzUxMiJ9.eyJpZCI6IjllNDk4YmUyLTZlZTEtNGJhOS04ZDdlLWFjZWRiOTk5ZWJkMCIsImRhdGEiOnt9LCJyYW5kb20iOiJlM2Q2MzlhNzY2ZmFmYWM1ZWRhMTYwYzk4NWRjYWYyMyJ9.8Hx0rmMI7eS0mhM2ONgxy-AgrG4LSWIyMdjQDNPxA4JbpgOQ-oT7jpAmVTKYbggWHVVRTbYVJUuDOhU8szCL0Q.
