# Supplementary material for: Comparative assessment of annotation tools reveals critical antimicrobial resistance knowledge gaps in Klebsiella pneumoniae
Source: Sci Rep. 2025 Nov 18;15:40495. doi: 10.1038/s41598-025-24333-9 (PMC12627748; doi:10.1038/s41598-025-24333-9)
Supplement: Supplementary file 2 — Supplementary Material 2 [file 41598_2025_24333_MOESM2_ESM.docx]

**Supplementary materials**

**Comparative assessment of annotation tools reveals critical antimicrobial resistance knowledge gaps in *Klebsiella pneumoniae***

*Kristina Kordova^1^, Caitlin Collins^1,2,3^, Julian Parkhill^1^*

*1.Department of Veterinary Medicine, University of Cambridge, Cambridge, UK*

*2.​UK Health Security Agency, London, UK*

*3.NIHR Health Protection Research Unit in Genomics and Enabling Data, University of Warwick, Warwick, UK*

*Email Address of the Corresponding Author: kvk22@cam.ac.uk*


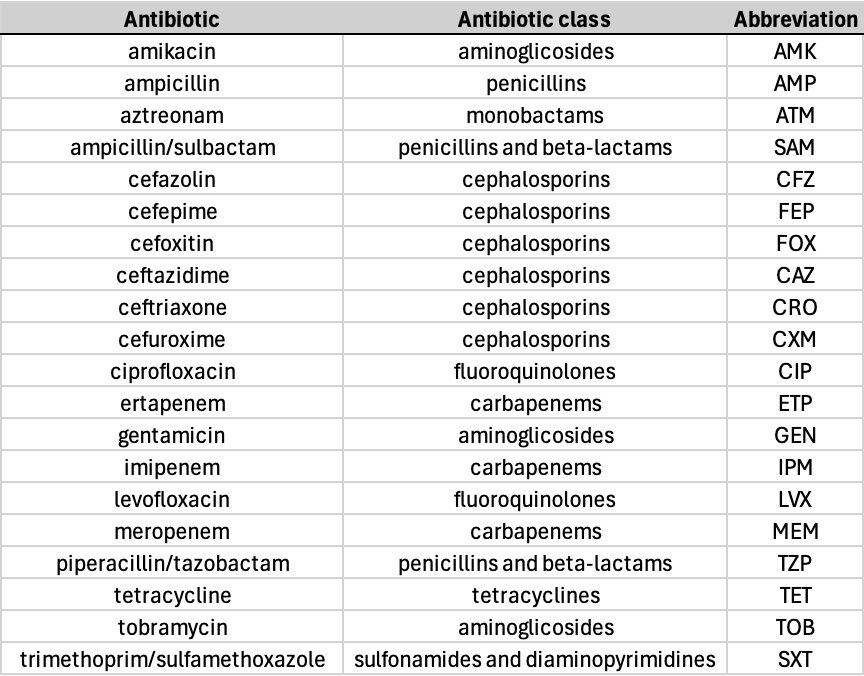


*Supplementary Table 1: List of antibiotics and their classes, used to match them against annotation tools, and their abbreviations.*

**
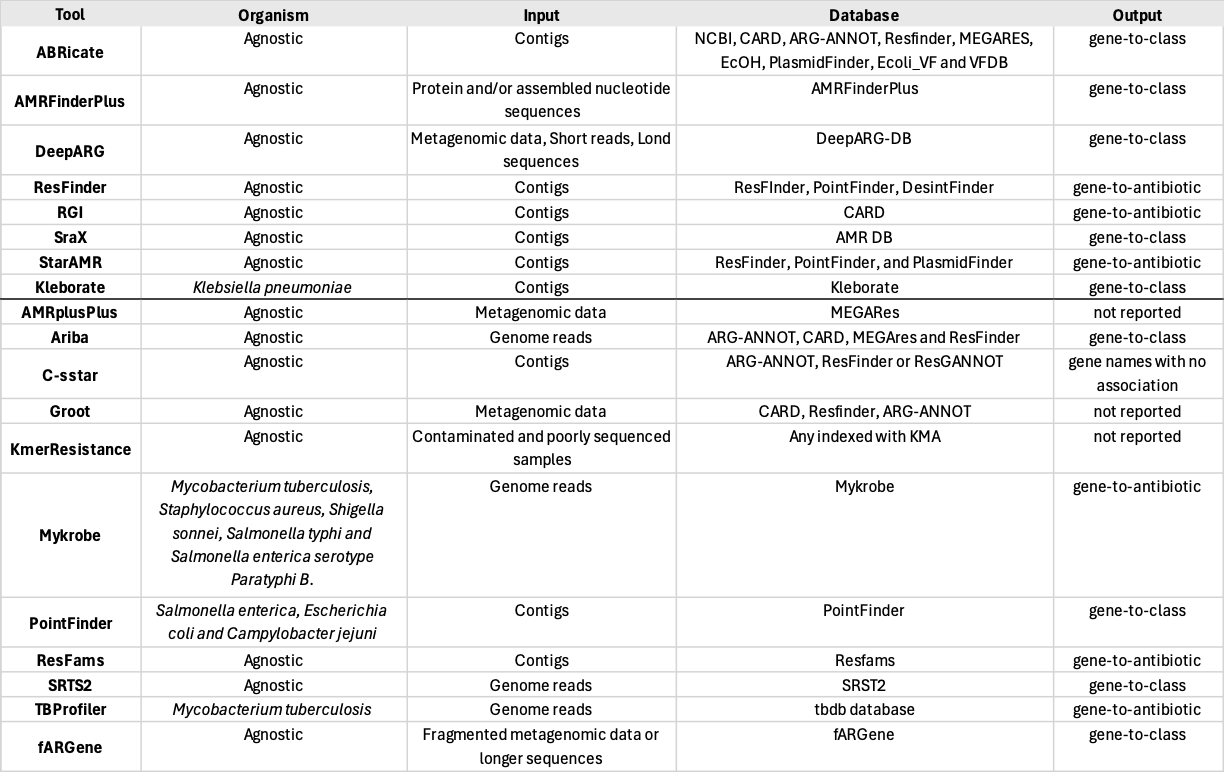
**

*Supplementary Table 2: List of the 19 AMR gene annotation tools identified for this analysis and description of their input, default databases and output formats. The eight tools which were compared in this analysis are listed above the bold divider.*


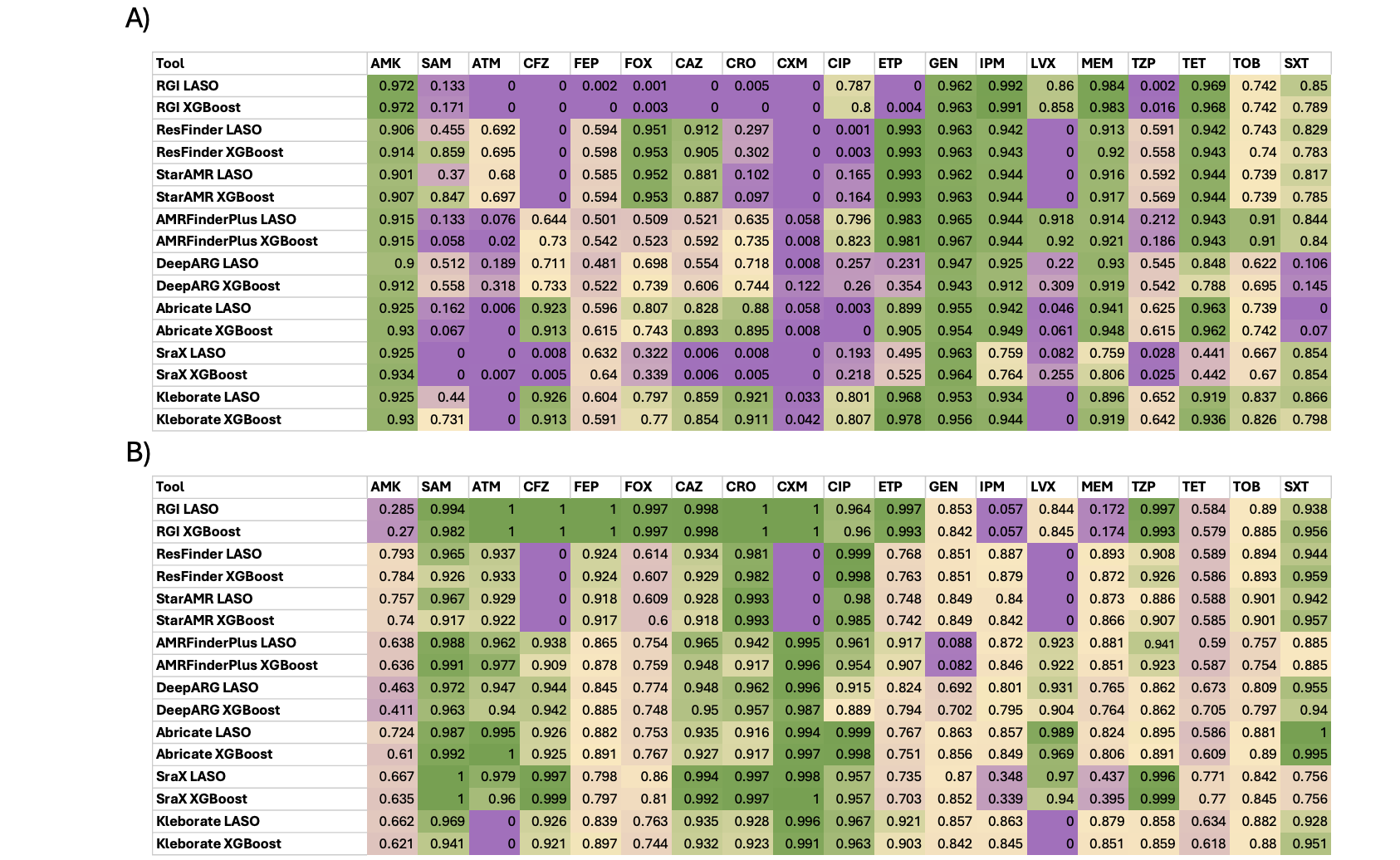


*Supplementary Table 3: A) Specificity and B) Sensitivity of phenotype predictions across antibiotics and annotation pipelines. Gene-to-antibiotic predictions are placed above the bold table line, and gene-to-class predictions are placed below. Ampicillin (AMP) performance sensitivity and specificity are determined by all samples being resistant to this antibiotic.*

[*Supplementary Table 4.xlsx*](https://zenodo.org/records/15755816/files/Supplementary%20Table%204.xlsx?download=1)

*Supplementary Table 4: Shapley values across tools and antibiotics. The displayed values represent the mean of the absolute values for each feature across all samples.*

*
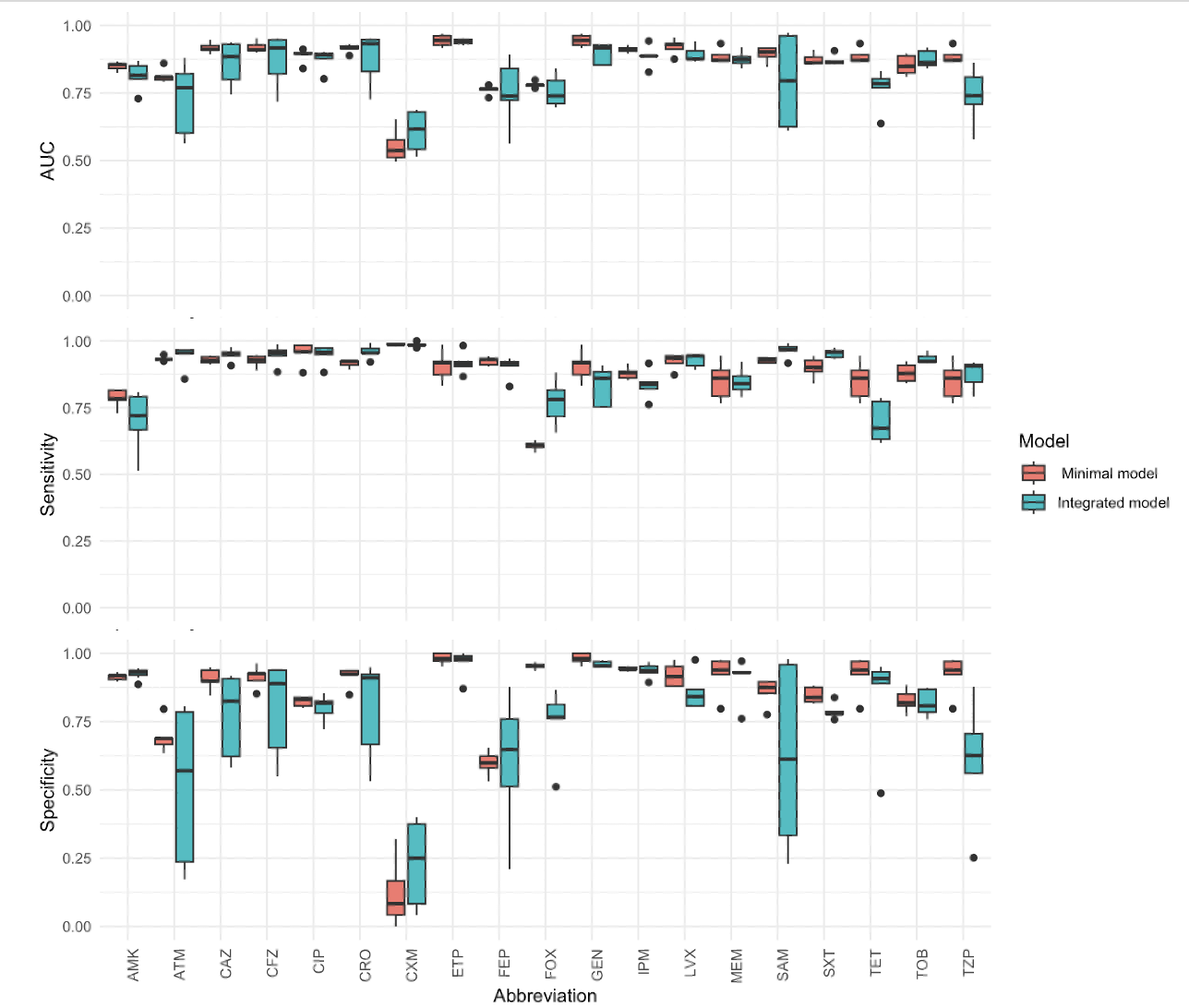
*

*Supplementary Figure 1: Comparison of the AUC, Sensitivity and Specificity of the top minimal model and the integrated feature model per antibiotic.*


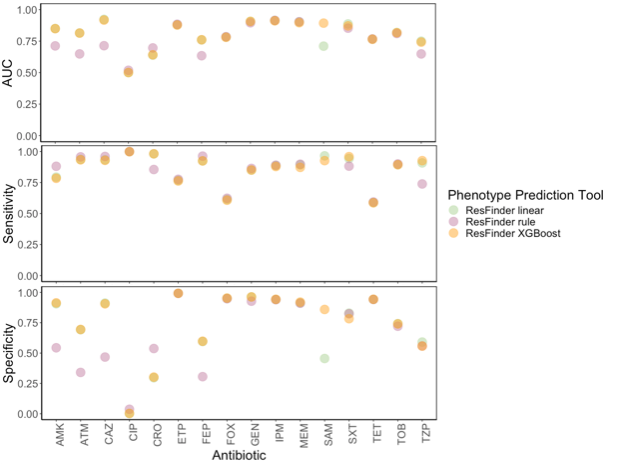


*Supplementary Figure 2: Comparison of the AUC, Sensitivity and Specificity of ResFinder linear, XGBoost and the pipeline’s native rule based phenotype prediction across antibiotics.*
